# Natalizumab Exerts Direct Signaling Capacity and Supports a Pro-Inflammatory Phenotype in Some Patients with Multiple Sclerosis

**DOI:** 10.1371/journal.pone.0052208

**Published:** 2012-12-20

**Authors:** Thomas F. Benkert, Lena Dietz, Elena M. Hartmann, Ellen Leich, Andreas Rosenwald, Edgar Serfling, Mathias Buttmann, Friederike Berberich-Siebelt

**Affiliations:** 1 Department of Molecular Pathology, University of Würzburg, Würzburg, Germany; 2 Institute of Pathology, University of Würzburg, Würzburg, Germany; 3 Department of Neurology, University of Würzburg, Würzburg, Germany; University of Palermo, Italy

## Abstract

Natalizumab is a recombinant monoclonal antibody raised against integrin alpha-4 (CD49d). It is approved for the treatment of patients with multiple sclerosis (MS), a chronic inflammatory autoimmune disease of the CNS. While having shown high therapeutic efficacy, treatment by natalizumab has been linked to progressive multifocal leukoencephalopathy (PML) as a serious adverse effect. Furthermore, drug cessation sometimes induces rebound disease activity of unknown etiology. Here we investigated whether binding of this adhesion-blocking antibody to T lymphocytes could modulate their phenotype by direct induction of intracellular signaling events. Primary CD4^+^ T lymphocytes either from healthy donors and treated with natalizumab *in vitro* or from MS patients receiving their very first dose of natalizumab were analyzed. Natalizumab induced a mild upregulation of IL-2, IFN-γ and IL-17 expression in activated primary human CD4^+^ T cells propagated *ex vivo* from healthy donors, consistent with a pro-inflammatory costimulatory effect on lymphokine expression. Along with this, natalizumab binding triggered rapid MAPK/ERK phosphorylation. Furthermore, it decreased CD49d surface expression on effector cells within a few hours. Sustained CD49d downregulation could be attributed to integrin internalization and degradation. Importantly, also CD4^+^ T cells from some MS patients receiving their very first dose of natalizumab produced more IL-2, IFN-γ and IL-17 already 24 h after infusion. Together these data indicate that in addition to its adhesion-blocking mode of action natalizumab possesses mild direct signaling capacities, which can support a pro-inflammatory phenotype of peripheral blood T lymphocytes. This might explain why a rebound of disease activity or IRIS is observed in some MS patients after natalizumab cessation.

## Introduction

Multiple sclerosis (MS) is a chronic inflammatory demyelinating disease of the CNS [1]. It may take a relapsing-remitting or a chronic progressive clinical course. The immigration of activated T lymphocytes into the CNS is fundamental to its pathogenesis [2]. During disease development, CD4^+^ T cells encounter environmental triggers of unknown kind in the periphery. This, in a widely accepted view, leads to activation of CNS antigen specific CD4^+^ T cells in genetically susceptible individuals. These autoreactive T cells then cross the blood-brain barrier (BBB) as effector T helper (Th) cells and initiate a chronic autoimmune disease [3].

Th1 cells, as defined by IFN-γ and TNF-α production, were found to be the predominant subpopulation of T lymphocytes in the CNS and the peripheral blood of MS patients [4], [5]. Later on, a newly defined lineage of T cells, named Th17 according to their signature lymphokine IL-17, could be linked to the development of experimental autoimmune encephalomyelitis (EAE), an animal model of MS [6]. Several recent publications considered Th17 cells, which additionally adopt the production of IFN-γ, as disease-promoting cells in EAE and also in MS [7].

Effector lymphocytes express elevated levels of adhesion molecules, enabling them to leave the vascular bed across the endothelium. More specifically, their firm arrest to endothelial cells is mediated by integrins. Integrin receptors are composed of non-covalently linked α- and β-chains. A special feature of integrins is their ability of bi-directional signaling [8]. The ‘inside-out’ signal is often evoked by chemoattractants and chemokines, but also by the T cell receptor (TCR), and induces integrin clustering as well as conformational changes, thereby enhancing avidity and/or affinity of an integrin receptor to its ligand [9]. Subsequent binding of the ligand initiates ‘outside-in’ signaling, which connects to growth factor pathways through protein-protein interactions [10].

The integrin α-4 (CD49d) is known to dimerize with either integrin β-1 (CD29) or β-7. These integrin subunits are expressed by *ITGA4*, *ITGB1* and *ITGB7*, respectively. The α-4/β-1 heterodimer – also termed VLA-4– allows firm arrest of lymphocytes to the vessel wall through engagement of its endothelial counterpart VCAM-1 (CD106). VCAM-1 is upregulated on brain microvascular endothelial cells in inflammatory CNS lesions of patients with MS [11], [12]. VLA-4 is strongly expressed on activated leukocytes including myelin basic protein (MBP) specific CD4^+^ T cells, which may trigger EAE but – such as any other antigen-specific T cell – are of unknown relevance to the human disease [13]. VLA-4 was found to be their main homing molecule for extravasation across the blood-brain barrier (BBB) into the inflamed CNS of EAE mice [14].

**Table 1:** List of “Top Ten Pathways” that were shown to be enriched in the group of natalizumab-treated CD4^+^ T cells.

Gene set	# genes ingene set (K)	# genes inoverlap (k)	k/K	P value
LEE_TCELLS3_UP	106	16	0.159	7.76E-06
MOREAUX_TACI_HI_VS_LOW_UP	422	37	0.0877	1.52E-05
GOLDRATH_CELLCYCLE	34	8	0.2353	6.11E-05
HSA04060_CYTOKINE_CYTOKINE_RECEPTOR_INTERACTION	257	24	0.0934	1.61E-04
NUCLEAR_RECEPTORS	40	8	0.2	2.00E-04
STEMPATHWAY	15	5	0.3333	2.68E-04
IDX_TSA_UP_CLUSTER3	90	12	0.1333	3.10E-04
CDC25PATHWAY	9	4	0.4444	3.29E-04
DOX_RESIST_GASTRIC_UP	44	8	0.1818	3.88E-04
GAMMA-UV_FIBRO_UP	35	7	0.2	4.92E-04

Genes were annotated to pathways (C2) using GSEA (Gene Set Enrichment Analysis) database, which showed at least a 0.5 fold [log2] stronger expression in the group of natalizumab-treated CD4^+^ T cells than in the group of natalizumab-untreated cells according to the respective mean values of three biological replicates.

**Table 2:** Natalizumab supports expression of cytokines and their receptors.

IL2	Interleukin 2
IL4	Interleukin 4
IL9	Interleukin 9
IL3	Interleukin 3 (colony-stimulating factor, multiple)
CSF1	Colony stimulating factor 1 (macrophage)
LTB	Lymphotoxin beta (TNF superfamily, member 3)
LTA	Lymphotoxin alpha (TNF superfamily, member 1)
CXCL5	Chemokine (C-X-C motif) ligand 5
CCL4	Chemokine (C-C motif) ligand 4
CCL1	Chemokine (C-C motif) ligand 1
CXCR3	Chemokine (C-X-C motif) receptor 3
IL17AR	Interleukin 17A
ACVR1B	Activin A receptor, type IB
FLT1	Fms-related tyrosine kinase 1
TNFRSF13C	Tumor necrosis factor receptor superfamily, member 13C
BMPR2	Bone morphogenetic protein receptor, type II
IL22	Interleukin 22
OSM	Oncostatin M
IL21	Interleukin 21
XCL1	Chemokine (C motif) ligand 1
FLT3LG	Fms-related tyrosine kinase 3 ligand
CCL28	Chemokine (C-C motif) ligand 28
XCL2	Chemokine (C motif) ligand 2
TNFSF14	Tumor necrosis factor (ligand) superfamily, member 14

Genes of the HSA04060_CYTOKINE_CYTOKINE_RECEPTOR_INTERACTION pathway are given which were found to be enriched in samples of natalizumab-treated cells by GSEA pathway annotation. Listed genes exhibited an at least 0.5-fold [log 2] stronger expression in natalizumab-treated than in natalizumab-untreated cells.

Natalizumab is a humanized monoclonal IgG4κ antibody directed against the human integrin α-4 subunit of VLA-4 and was generated to physically interfere with lymphocyte extravasation across the BBB in patients with MS, after the central pathogenic relevance of VLA-4 in EAE had been discovered [15]. In patients with relapsing-remitting MS (RRMS), natalizumab reduced the annualized relapse rate by 68% compared to placebo, caused a 42% reduction of sustained disability progression over two years, and had a strong therapeutic effect on MRI disease activity [16]. It is approved for the treatment of patients with RRMS in Europe and the US. 104,300 patients worldwide had received the drug since its approval until June 30^th^, 2012. Natalizumab is well tolerated by most MS patients. However, its use is limited by the rare occurrence of progressive multifocal leukoencephalopathy (PML), a life-threatening opportunistic brain infection by JC virus [17]. As of October 3^rd^ 2012, natalizumab-associated PML affected 298 individuals, corresponding to ∼2.71 in 1,000 patients, 63 (21%) of whom had died until that time point. The risk of PML independently increases with treatment duration and immunosuppressive pretreatment. Furthermore, it is determined by the JCV serostatus, which indicates a potential latent JCV infection along with a risk of PML when positive, while the risk is <1∶5.000 when the test is negative [17], [18].

To rapidly remove natalizumab in case of PML, patients receive plasma exchange or immunoabsorption to hasten immune reconstitution. However, they regularly develop exuberant CNS inflammatory disease activity a few weeks later, termed immune reconstitution inflammatory syndrome (IRIS) or more specifically PML-IRIS [19], [20], [21], [22]. Without PML, MS disease activity after natalizumab cessation most often returns to pretreatment levels within a few months [23]. However, cases of rebound disease activity were observed by a number of investigators and in our own clinical experience [24], [25], [26], [27]. This occasional post-treatment overshoot of CNS inflammation might partially be due to an increased percentage of pro-inflammatory CD4^+^ and CD8^+^ T cells, as observed in the peripheral blood during treatment with natalizumab over six months and longer [28], [29], [30]. Following natalizumab cessation, inflammatory activated lymphocytes may then enter the CNS, which is no longer protected by the drug.

The reason for the occurrence of peripheral pro-inflammatory T lymphocytes during natalizumab treatment is unknown. Therefore, we decided to explore the capability of this anti-integrin α-4 antibody to modulate the phenotype of T lymphocytes by direct induction of intracellular signaling. And indeed, natalizumab, designed to block CD49d-mediated immune cell extravasation, was found to induce overall mild ‘outside-in’ signals in activated T lymphocytes along with an increased expression of pro-inflammatory cytokines. In line with inter-individual differences with respect to rebound disease and PML-IRIS, not all T cells were equally responsive. Therefore, natalizumab signals directly and – dependent on the immunological situation of individual patients with MS – can serve as a pro-inflammatory costimulus for T cell receptor-activated cells.

## Materials and Methods

### Subjects

All experiments involving MS patients were approved by the Ethics Committee of the Faculty of Medicine at the University of Würzburg. Written informed consent was obtained from all 9 patients prior to collection of blood samples. All of them had RRMS according to the 2005 revision of the McDonald diagnostic criteria [31]. Eight patients were female, one was male. Median age at study inclusion was 32 (range 23–46) years. Median disease duration was 7 (range 2–15) years. Median Expanded Disability Status Scale (EDSS) at study inclusion was 3.0 (range 1.5–4.0). All patients had experienced break-through disease activity on secondary prophylactic treatment with an approved interferon β preparation or glatiramer acetate before starting natalizumab therapy. Three of 9 patients had received immunosuppressive treatment with mitoxantrone and/or azathioprine in the past. None of the patients had received corticosteroid treatment within 3 weeks before study inclusion and none had an acute relapse. Blood was taken immediately before the very first infusion of 300 mg natalizumab (Tysabri®, Biogen Idec/Elan, Ismaning, Germany) intravenously in a routine clinical setting and 24 h after infusion.

Blood from healthy donors of random age and gender was obtained after written informed consent in accordance with the Declaration of Helsinki, under a protocol that had received Institutional Review Board approval from the Ethics Committee of the Faculty of Medicine at the University of Würzburg. In addition, PBL generated during thrombocytapheresis of blood from healthy donors were kindly provided by the local Department of Transfusion Medicine. Since these blood samples were offered anonymously, the Ethics Committee of the Faculty of Medicine at the University of Würzburg gives a general approval.

### Cell Isolation, Cultivation and Stimulation

PBMC were isolated from EDTA blood samples via centrifugation in Biocoll® separating solution (Biochrom, Berlin, Germany). CD4^+^ T lymphocytes were enriched by positive selection using a MACS human CD4 microbeads isolation kit (Miltenyi Biotech, Bergisch Gladbach, Germany). Purity of the CD4^+^ population was typically >85% up to 95%.

For microarray gene expression profiling, 3.0×10^5^ CD4^+^ T cells were placed in 25 ml CTL medium containing 30 Gy irradiated PBMC (2.5×10^7^), 60 Gy irradiated T2 feeder cells (5.0×10^6^), and OKT-3 (30 ng/ml) for 3 days [32]. Recombinant IL-2 (50 U/ml) was added on days 1, 3, 7 and 10. After this expansion and synchronization, cells were reseeded either in the absence or presence of natalizumab (30 µg/ml) on day 13. The natalizumab concentration was chosen based on mean average steady-state through serum levels of 23 to 29 µg/ml and mean peak serum levels of 110±52 (SD) µg/ml in MS patients receiving 300 mg natalizumab every 28 days, as reported in the prescribing information. Natalizumab (Tysabri®) for *in vitro* experiments was purchased from our local pharmacy at the University Hospital Würzburg. Ten days after reseeding, cells were restimulated with T/I (10 ng/ml TPA, Sigma-Aldrich, Hamburg, Germany; 2 µM ionomycin, Life Technologies, Darmstadt, Germany) for 8 h and fresh natalizumab was added in order to recall the developed program and induce cytokine expression. Subsequently, cells were pelleted and resuspended in 1 ml Trizol® (Life Technologies).

Except for microarray experiments, PBMC and CD4^+^ T lymphocytes (1–5×10^6^ cells/ml) were cultured in complete X-VIVO15 medium, supplemented with 10% heat-inactivated, pooled human AB serum and 100 U/ml penicillin-streptomycin (all from Life Technologies). Jurkat cells (5×10^6^ cells/ml) were kept in complete RPMI1640 medium, containing 5% heat-inactivated FCS. Cells were stimulated for the indicated times (2, 4, 8, 24, 48, 72 h): 1 µg/ml staphylococcus enterotoxin B (SEB, Toxin Technology, Sarasota, FL), anti-CD3/28-Dynabeads® (Life Technologies) or plate-bound anti-CD3/28 mAb (both 5 µg/ml, BD Biosciences, Heidelberg, Germany), T/I (10 ng/ml/2 µM) in the absence or presence of natalizumab (30 µg/ml), controlled by IgG4 (BD Biosciences or Sigma Aldrich) or HP2/1 (Abcam, Cambridge, UK; Abnoby, Heidelberg, Germany; or Beckman Coulter, Krefeld, Germany). For intracellular FACS stainings 1 µg/ml brefeldin A or brefeldin A plus 1.34 µg/ml monensin (for IL-17 and IFN-γ was added for the last 8–12 h.

### Gene Expression Arrays

Gene expression profiling was performed with HG U133 plus 2.0 gene expression arrays (Affymetrix) following the Affymetrix expression analysis technical manual (www.affymetrix.com). Data were normalized by the Gene Chip Operating Software (GCOS) from Affymetrix. The intensity values of each donor were averaged in groups and the mean values of the natalizumab samples subtracted from those without natalizumab treatment. Sorted intensity values of stimulated and non-stimulated samples in an ascending and descending manner determined genes with the most prominent and at least two-fold differences in expression. For pathway annotation (at least 0.5-fold [log2] change) a two-tailed t-test and gene set enrichment analysis (GSEA) (http://www.broad.mit.edu/gsea) was performed. For cluster analysis and visualization of the data the Cluster and TreeView software packages (http://rana.lbl.gov/EisenSoftware.htm, M. Eisen, Berkeley, CA) were used.

### FACS Staining

Cells were harvested, washed in PBS and FACS buffer (PBS containing 0.1% BSA), blocked with Fc receptor blocking reagent (BD Biosciences) for 10 min at room temperature (RT) and rinsed with FACS buffer. Surface staining was performed using anti-human CD4-FITC and anti-human CD49d-PE (all BD Biosciences), for 30 min at RT, and fixation with 2% paraformaldehyde (PFA) in PBS, for 20 min at RT. For intracellular staining cells were incubated in permeabilization buffer (FACS buffer containing 0.1% saponin), for 10 min at RT, pelleted, resuspended in a small volume of permeabilization buffer, and stained with anti-IFN-γ-FITC, anti-IL-17-APC or anti-IL-2-APC (all Miltenyi Biotech), for 90 min at 4°C. Washed samples were measured on the FACS LSRII flow cytometer (BD Biosciences) and analyzed using FlowJo software (Tree Star, Ashland, OR, http://www.flowjo.com).

### Quantitative RT-PCR

Total RNA from human primary CD4^+^ T cells was purified using Trizol® (Life Technologies), and cDNA was accomplished by the iScript cDNA synthesis kit (BioRad, Hercules, CA). Quantitative RT-PCR (qRT-PCR) was performed by using an ABI 7000 RT-PCR instrument (Applied Biosystems, Carlsbad, CA). All primers were TaqMan® probes from Applied Biosystems and used according to the manufacturer’s standard protocols. Normalization for each sample was achieved by using the formula: ΔCT = CT_(target gene)_ – CT_(B2M)_. Relative expression was calculated with the formula: 2^–ΔCT^.

### Immunoblot

Whole cellular protein lysates were made from 2×10^7^ Jurkat or CD4^+^ T cells using RIPA buffer and the snap-freezéńthaw method. Nuclear and cytosolic protein extracts were generated using the ProteoJET® Cytoplasmic and Nuclear Protein Extraction Kit (Fermentas Life Sciences, St. Leon-Rot, Germany). An equal amount of total protein was fractionated by 12% SDS-PAGE and electroblotted onto nitrocellulose membrane. For detection, anti-JNK, anti-pJNK, anti-ERK, anti-pERK and anti-NFATc1 antibodies (all from Santa Cruz Biotechnology, Heidelberg, Germany), with corresponding anti-mouse or anti-rabbit peroxidase-coupled secondary antibodies (Sigma Aldrich) were used with an enhanced chemiluminescence system (Pierce, Rockford, IL). To analyze CD49d protein levels in cell culture supernatants, proteins were first concentrated by centrifugation of 300 µl per stimulation condition supernatant through Microcon YM-10 centrifugal filter devices (Millipore, Schwalbach/Ts., Germany). The recovered proteins were fractionated by 8% SDS-PAGE and electroblotted onto nitrocellulose membrane. For detection, two different mAbs against the extracellular domain of CD49d (clone L25, BD Biosciences; clone HP2/1, Beckman Coulter) were used with an anti-mouse peroxidase-coupled secondary antibody (Jackson ImmunoResearch via Dianova, Hamburg, Germany) along with an enhanced chemoluminescence system (BD Biosciences).

### Cytokine Measurement

PBMC from MS patients were isolated, resuspended at a total of 5×10^6^/ml cells and stimulated with plate-bound anti-CD3/28 (both BD Biosciences) in a 24-well tissue culture plate. Supernatants were collected after 48 h and cytokine measurement of human IFN-γ, IL-2, IL-10, IL-4, IL-21, IL-12/IL-23 p40 and IL-17 was performed using a cytometric bead array (CBA) kit (BD Biosciences). The mixtures were incubated for 3 h; beads were washed and analyzed using flow cytometry (FACS CantoII, BD Biosciences). Cytokine concentration (pg/ml) was calculated using CBA software.

### Immunocytochemistry

Following stimulation as indicated, 5×10^4^ PBMC per stimulation condition were transferred to microscope slides, using a standard cytospin protocol. Cells were fixed with 3.7% PFA for 10 min and permeabilized with 0.1% Triton X-100 for 5 min at RT, washed two times with phosphate-buffered saline and blocked in PBS containing 5% BSA for 1 h at RT. Subsequently, a primary mouse mAb against CD49d (clone L25) in PBS containing 1% BSA was incubated at 4°C overnight. Then, a Cy3-coupled anti-mouse secondary antibody (Jackson ImmunoResearch) was incubated for 1 h at RT. Nuclei were counterstained with DAPI. Finally, an antifading agent comprised of Mowiol 4–88 (Calbiochem), glycerole and Dabco (Sigma Aldrich) in 0.2 M Tris HCl was added. Negative controls were performed by staining with an isotype-matched IgG2b control antibody (BD Biosciences). The stainings were analyzed by confocal laser scanning microscopy (Leica TCS SP2 equipment, objective lense; HeX PL APO, 40×/1.25–0.75) and LCS software (Leica).

### ELISA

Total protein levels of matrix metalloproteinases MMP-2 and MMP-9 in culture supernatants of 1×10^7^ PBMC in 2 ml complete X-VIVO15 medium as detailed above were measured using commercial ELISA kits (R&D, Wiesbaden-Nordenstadt, Germany) according to the instructions of the manufacturer. Both MMP-2 and MMP-9 were detectable in all supernatants. All samples were analyzed in duplicates. Mean intraassay reproducibility was 7.4% for MMP-2 and 6.2% for MMP-9.

### Statistical Analysis

Except for pathway annotation of microarray data, where two-tailed t tests were employed (see above), non-parametric tests were used throughout the study. For comparison of two conditions, a two-tailed Wilcoxon matched-pairs signed rank test or a two-tailed Mann-Whitney test were employed as appropriate. For comparison of more than two time points, Bonferroni adjustment was applied, when indicated. Alternatively, a two-tailed Friedman test or a two-tailed Kruskal-Wallis test were performed, which both were followed by Dunn’s multiple comparisons test, if p values were <0.05. For correlation analysis, Spearman tests were used. A p value <0.05 was considered as statistically significant. GraphPad PRISM 5 software (La Jolla, CA) was used for all analyses.

## Results

### The Blocking Antibody Natalizumab Signals

**Figure 1:**
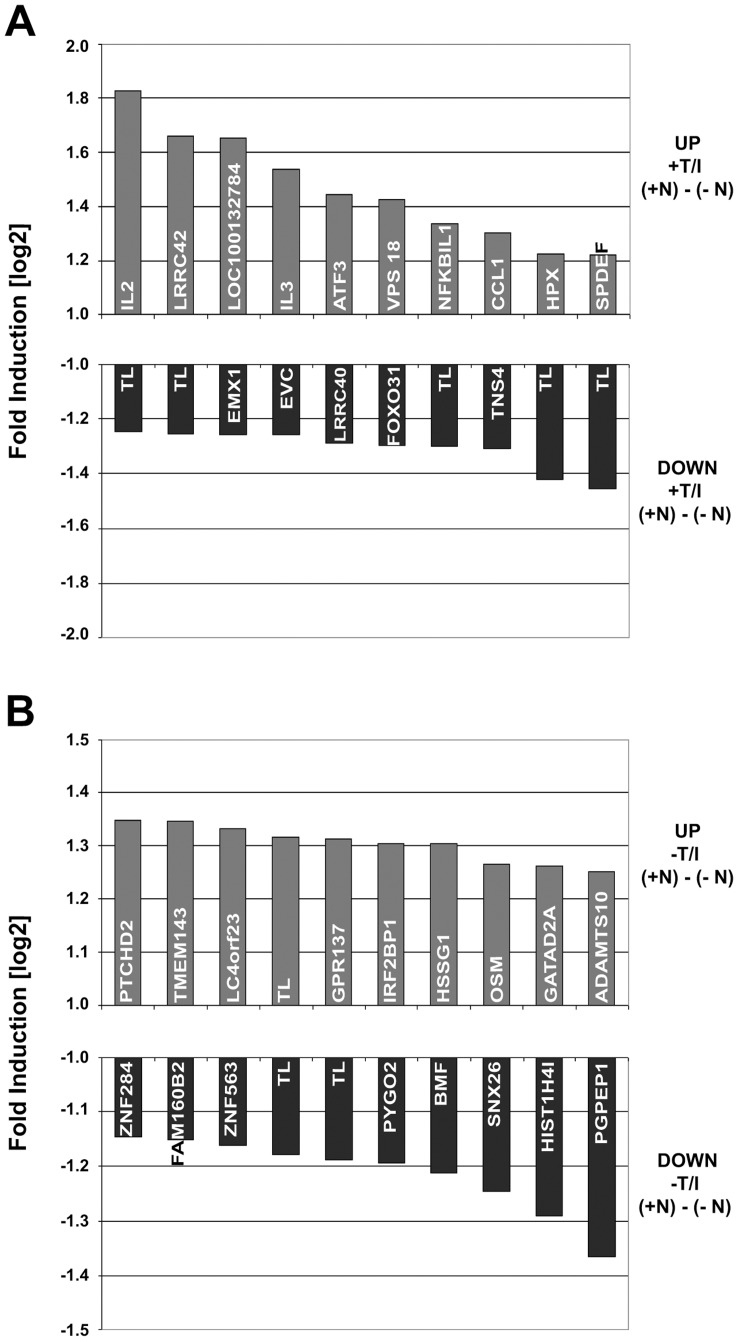
Top ten genes with natalizumab-driven differential expression levels. (A) List of genes that were influenced the most by natalizumab in CD4^+^ T cells, which were restimulated for 8 h. (B) List of genes that were influenced the most by natalizumab in CD4^+^ T cells, which were not restimulated.

In order to detect any possible signaling via natalizumab we performed an unbiased screen by gene expression profiling using the HG U133 plus 2.0 gene expression arrays (Affymetrix). RNA was prepared from CD4^+^ T cells grown and (re-) stimulated in the presence or absence of natalizumab. The Affymetrix expression analysis revealed only mild changes. Nevertheless, several pathways were – positively or negatively – affected, clearly demonstrating a signaling potential of natalizumab (Table 1). The most prominently influenced gene encoded IL-2 with an approximately 3.5-fold (linear) upregulation by natalizumab (Figure 1A, B). Expression differences of immunologically relevant genes became evident by the Gene Set Enrichment Analysis (GSEA) pathway annotation (Table 2). Specifically, we observed the enrichment of a cytokine-cytokine receptor interacting pathway among the ten most significantly influenced pathways in the group of natalizumab-treated compared to untreated samples.

### Natalizumab Enhances IL-2 and Th17-specific Lymphokines

**Figure 2:**
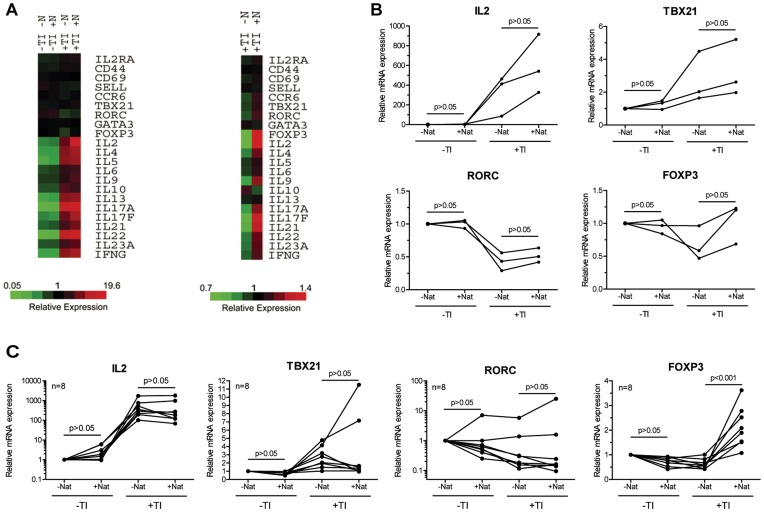
Natalizumab supports a pro-inflammatory gene expression profile. (A) RNA expression differences of three independent biological replicates are depicted in heat maps for immunologically relevant genes. Cells had not or had been restimulated with T/I (TI). Both groups had been grown with or without natalizumab (N or Nat). (B) qRT-PCR of RNA used for the microarray (triplicates) was analyzed for RNA levels of IL-2 as well as RORγt, T-BET and FOXP3. These three transcription factors are transcribed from the genes *RORC*, *TBX21* and *FOXP3*, respectively. (C) Independent replication of the qRT-PCR results in 8 different additional donors. Here, CD4^+^ T cells were stimulated by anti-CD3/28-Dynabeads® for 10 days and restimulated with T/I for 8 h. Dots represent single donors, lines connect intraindividual values. Friedman tests followed by Dunn’s post tests were performed in (B) and (C).

To analyze the effect of natalizumab on lymphokine expression more closely, important lymphokines, surface molecules, and the key regulators of CD4^+^ T cell differentiation were compared on a heat map (Figure 2A). The fold change was highest for IL-2, but other lymphokines were also positively influenced by natalizumab. Important in the context of MS, the panel of Th17-specific lymphokines was upregulated, whereas the inhibitory lymphokine IL-10 was repressed by natalizumab. RNA for the homing receptor of Th17, CCR6, was enhanced. Enrichment could also be detected for FOXP3, which is the key transcription factor in regulatory T cells, but also indicative of activation in human effector T cells [33]. RNA levels of the transcriptional key regulators of Th17 and Th1 cells, transcribed from *RORC* and *TBX21,* respectively, were upregulated through engagement of integrin α-4 by natalizumab. The differences in RNA expression were validated by qRT-PCR with the donors’ RNA samples used for the microarrays. Moderately increased expression of IL-2 and of all three tested key transcription factors under the influence of natalizumab was noted in all specimens from all 3 donors in this exploratory analysis (Figure 2B). Differences in the levels of transcription factor specific RNA turned out to be inter-individually regulated, when analyzing 8 additional donors under slightly altered experimental conditions. However, using a multiple comparison test plus Bonferroni correction for testing of 4 targets, mRNA for FOXP3 was enhanced by natalizumab with high significance, strongly supporting the notion of natalizumab to possess signaling capacities (Figure 2C).

### Natalizumab Exerts a Specific Direct Signaling Effect

**Figure 3:**
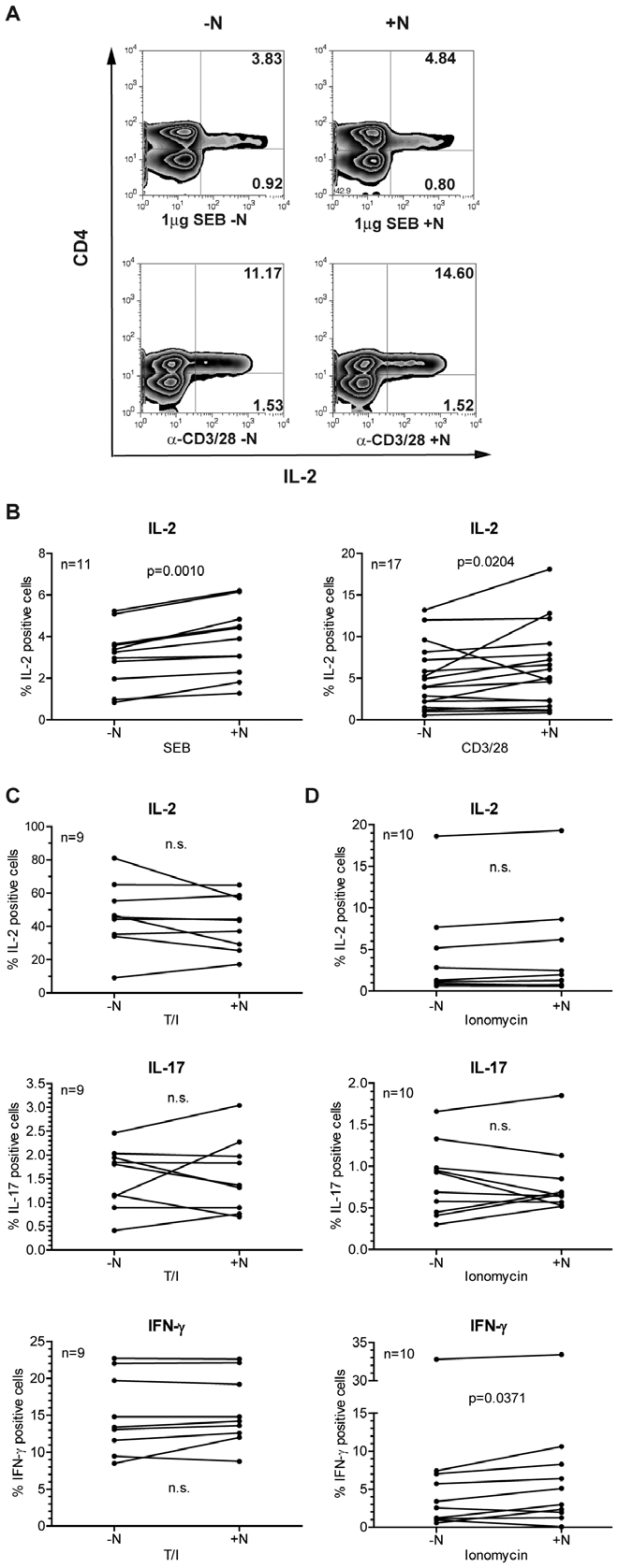
Intracellular protein levels of IL-2, IL-17 and IFN-γ are enhanced by concomitant treatment with natalizumab. (A, B) PBMC were stimulated by SEB or anti-CD3/28 without or in the presence of natalizumab for 24 h. After surface staining for CD4 and intracellular staining for IL-2, cells were analyzed by FACS. (A) Representative dot blots are given. (B) Single values of samples from n = 11 (SEB) and n = 17 (anti-CD3/28) donors are shown. (C, D) Isolated CD4^+^ T cells were stimulated with T/I (n = 9) or ionomycin (n = 10) for 8 h. Intracellular FACS stainings for IL-2, IL-17, and IFN-γ were performed, and the differences between cultures with and without natalizumab were calculated. Two-tailed Wilcoxon matched-pairs signed rank tests were performed for statistical analysis in (B, C, and D).

If natalizumab exerts a direct signaling effect, an influence should become detectable within the time frame of one cell cycle. And indeed, intracellular staining for IL-2 and concomitant FACS analyses demonstrated that CD4^+^ T cells stimulated with superantigen (SEB) or anti-CD3/28 for only 24 h both expressed significantly more IL-2 in the presence of natalizumab (Figure 3A, B).

In addition to IL-2, expression of IL-17 and IFN-γ under the influence of natalizumab was analyzed on the protein level. In order to achieve sufficient effector lymphokine expression, CD4^+^ T cells were activated with the chemical compounds TPA and ionomycin, bypassing, but mimicking TCR crosslinking by activating protein kinase C and Ca^2+^/calcineurin, respectively, for only 24 h. Intracellular FACS staining revealed robust IL-2 expression after T/I stimulation (median 45.6% CD49d^hi^ cells without presence of natalizumab), which was more than ten times higher than that mediated by SEB (median 3.2%) or anti-CD3/28 (median 4.0%) stimulation (Figure 3C). Natalizumab induced a significant alteration of cytokine levels with both of the weaker stimuli (Figure 3B), while no significant alteration was observed with T/I stimulation (Figure 3C). On the other hand, stimulation with ionomycin alone (median 1.2% CD49d^hi^ cells), which mimics a solely TCR crosslinking mediated Ca^+^ influx, although the levels of IL-2 and IL-17 were not significantly altered, allowed to detect a significant upregulation of IFN-γ expression (Figure 3D). Together, these data demonstrated a direct costimulatory potential of natalizumab via integrin α-4, which became obvious under conditions mimicking physiological T cell activation.

### Natalizumab Supports ERK Phosphorylation

**Figure 4:**
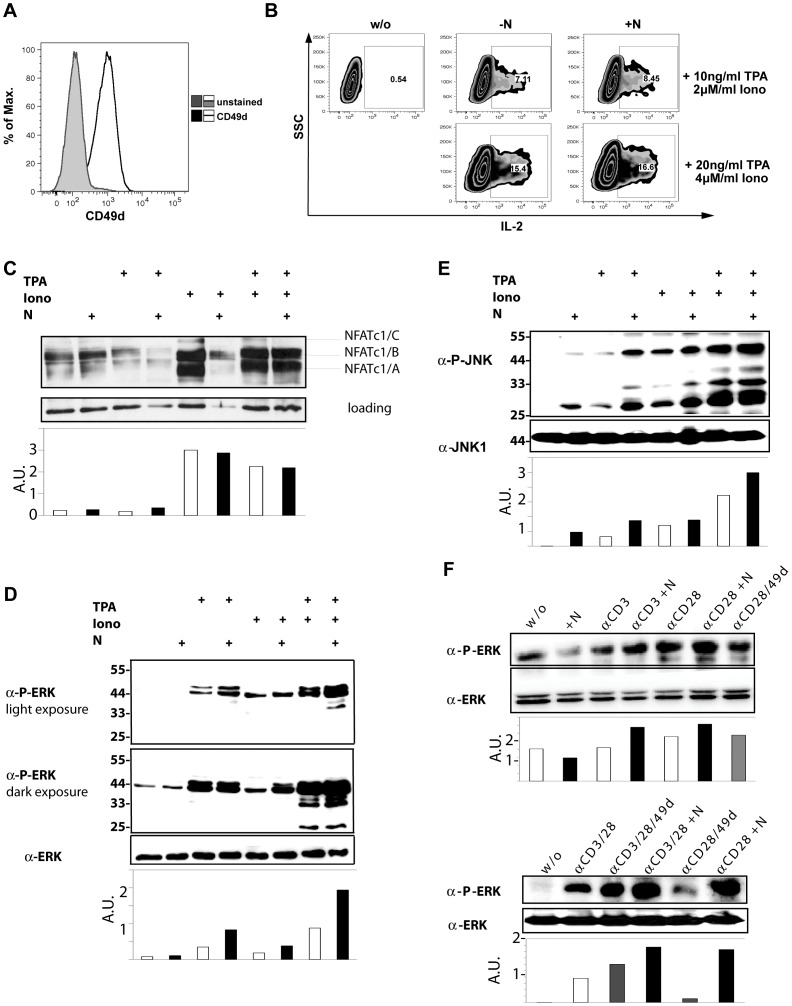
Natalizumab signals via rapid phosphorylation of ERK. (A) Surface expression of CD49d was verified for Jurkat cells. (B) Intracellular FACS staining for IL-2 revealed increased expression in the presence of natalizumab, when cells were stimulated by T/I (2 concentrations) for 8 h. (C) Western blot analysis of nuclear extracts for NFATc1 after stimulation as indicated for 2 h. (D, E) Immunoblot of proteins extracted from Jurkat cells after stimulation with TPA, ionomycin or both in the presence or absence of natalizumab for 2 h. (D) Detection of pERK and ERK within whole cell extracts. (E) Detection of pJNK and JNK within whole cell extracts. (F) Immunoblot of total proteins extracted from human CD4^+^ T cells after stimulation with anti-CD3/28– alone or in combination - in the absence or presence of either agonistic anti-CD49d or natalizumab for 2 h. Phosphorylated ERK and total ERK were detected.

If integrin α-4 can be triggered by natalizumab, footmarks of the induced signaling should be detectable. For molecular analyses, we initially used the human CD4^+^ T cell line Jurkat. Surface expression of integrin α-4 (Figure 4A) and a positive influence of natalizumab on IL-2 expression was verified for Jurkat T cells (Figure 4B). IL-2 and most other lymphokines are transcribed in dependency of NFAT activation, which is downstream of Ca^2+^/calcineurin signals and indicated by its translocation to the nucleus. Accordingly, we found an enrichment of the C3 motif gene set NFAT when we annotated the genes that were found to be upregulated (≥0.5 [log2]) in natalizumab-treated CD4^+^ T cells using GSEA. However, when NFAT activation was evaluated, no increased nuclear translocation under the influence of natalizumab could be observed (Figure 4C).

Thereafter, we analyzed MAPK pathways described to be touched by integrin signals. Jurkat T cells were stimulated by TPA and/or ionomycin. (i) While the overall expression of ERK was unchanged during natalizumab treatment (Figure 4D, lowest panel), phosphorylation could be rapidly enhanced by natalizumab during stimulation with either TPA or the combined action of T/I (Figure 4D, both upper panels). (ii) Phosphorylation of JNKs was supported by natalizumab in TPA-treated Jurkat T cells (Figure 4E), while any change in phosphorylation of p38 MAPK could not be detected (data not shown). In primary CD4^+^ T cells, the costimulatory effect of natalizumab for TCR crosslinking could be verified for ERK phosphorylation (Figure 4F), while it was barely detectable for JNK phosphorylation (data not shown). Unexpectedly, natalizumab even provided a better costimulatory signal than an agonistic anti-CD49d (clone HP2/1) antibody. Taken together, natalizumab’s signaling capacity resembled features of natural integrin signaling.

### Natalizumab-induced Reduction of CD49d Surface Expression Depends on T Cell Activation State

**Figure 5:**
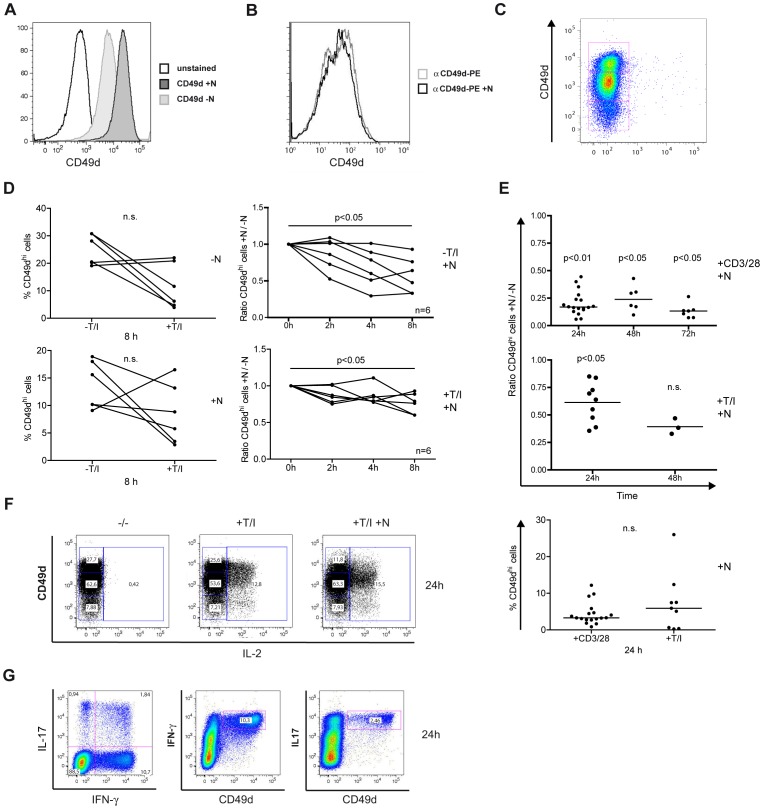
Natalizumab reduces CD49d surface expression. (A) Jurkat cells were treated with natalizumab for 24 h and stained for CD49d expression. (B) A possible interference of natalizumab and the staining antibody for CD49d was evaluated. (C) T/I-activated PBMC incubated with natalizumab for 24 h prior to staining and FACS analyses. Gated on CD4^+^ T cells, CD49d appeared in three subpopulations. (D) Percentage of CD49d^hi^ cells among all CD4^+^ cells after stimulation as indicated for 8 h (left column) and normalization of natalizumab-stimulated to natalizumab-unstimulated cells without and with T/I activation for 2, 4 and 8 h (right column). CD49d^hi^ expression was evaluated by FACS. (E) CD4^+^ T cells were activated with CD3/28 or T/I in the absence or presence of natalizumab for the indicated durations. Ratios of natalizumab-stimulated vs. unstimulated cells were calculated (upper two diagrams). Lower diagram shows percentage of CD49d^hi^ cells among all CD4^+^ cells after activation with CD3/28 or T/I for 24 h. Same samples as in first columns of upper and middle diagram. (F) CD4^+^ T cells had been preincubated for 24 h with natalizumab, finally stimulated by T/I for 8 h, stained for CD49d surface and IL-2 intracellular expression and evaluated by FACS. (G) CD4^+^ T cells were stimulated by T/I for 8 h and stained for intracellular IL-17 and IFN-γ as well as for surface expression of CD49d, analyzed by FACS.

A common feature of receptor engagement and signaling is the concomitant internalization. We sought to follow integrin α-4 expression on the surface of Jurkat T cells when solely natalizumab was added. Clearly, CD49d was reduced by the magnitude of roughly half a log scale within 24 h (Figure 5A). This was not due to competitive binding of natalizumab and the anti-CD49d antibody for FACS staining (Figure 5B). When CD4^+^ T cells were tested for their pattern of CD49d expression, three populations, with low, intermediate or high CD49d density, could be observed (Figure 5C).

Activation of T cells by T/I for 8 h (without natalizumab) strongly reduced the amount of CD49d^hi^ CD4^+^ T cells in 4 of 6 samples from independent donors in comparison to unstimulated cells (median 69.6% reduction, Figure 5D, left upper diagram). However, when natalizumab was given in addition, the ratio of CD49d^hi^ CD4^+^ T cells in +natalizumab vs. –natalizumab samples tended to decrease more rapidly and more pronounced with no further stimulation besides natalizumab addition (Figure 5D, right column). Nevertheless, both resting and T/I-activated T cells showed a significant relative reduction of CD49d^hi^ populations 8 h after natalizumab addition. Percentages of CD49d^hi^ cells were lower among all CD4^+^ T cells when treated with natalizumab (+T/I, median 7.3 vs. –T/I; 12.9%, Figure 5D, left lower diagram).

When comparing CD3/28- and T/I-stimulated cells over a longer period of time, the relative reduction of CD49^hi^ T cells due to natalizumab treatment over 24 h was much more pronounced after activation by CD3/28 costimulation vs. T/I treatment in this independent set of samples (Mann-Whitney p<0.0001, Figure 5E, compare first columns of upper and middle panel), while the percentage of CD49d^hi^ cells among all CD4^+^ cells did not differ between natalizumab-treated CD3/28- vs. T/I-activated cells (Figure 5E, lowest panel). We furthermore demonstrated that natalizumab-induced CD49d reduction was sustained and did not tend to increase over 72 h of CD3/28 activation (Figure 5E, upper panel).

Under all conditions, the CD49d^hi^ population was predominantly reduced upon natalizumab addition. Major IL-2 correlated with CD49d^hi^ expression and was only forced into low and intermediate integrin α-4 expressers by natalizumab (Figure 5F). Of note, the CD49d^hi^ population produced IFN-γ and IL-17 as well (Figure 5G).

### Integrin α-4 is Rapidly Internalized and Degraded Upon Natalizumab Engagement

**Figure 6:**
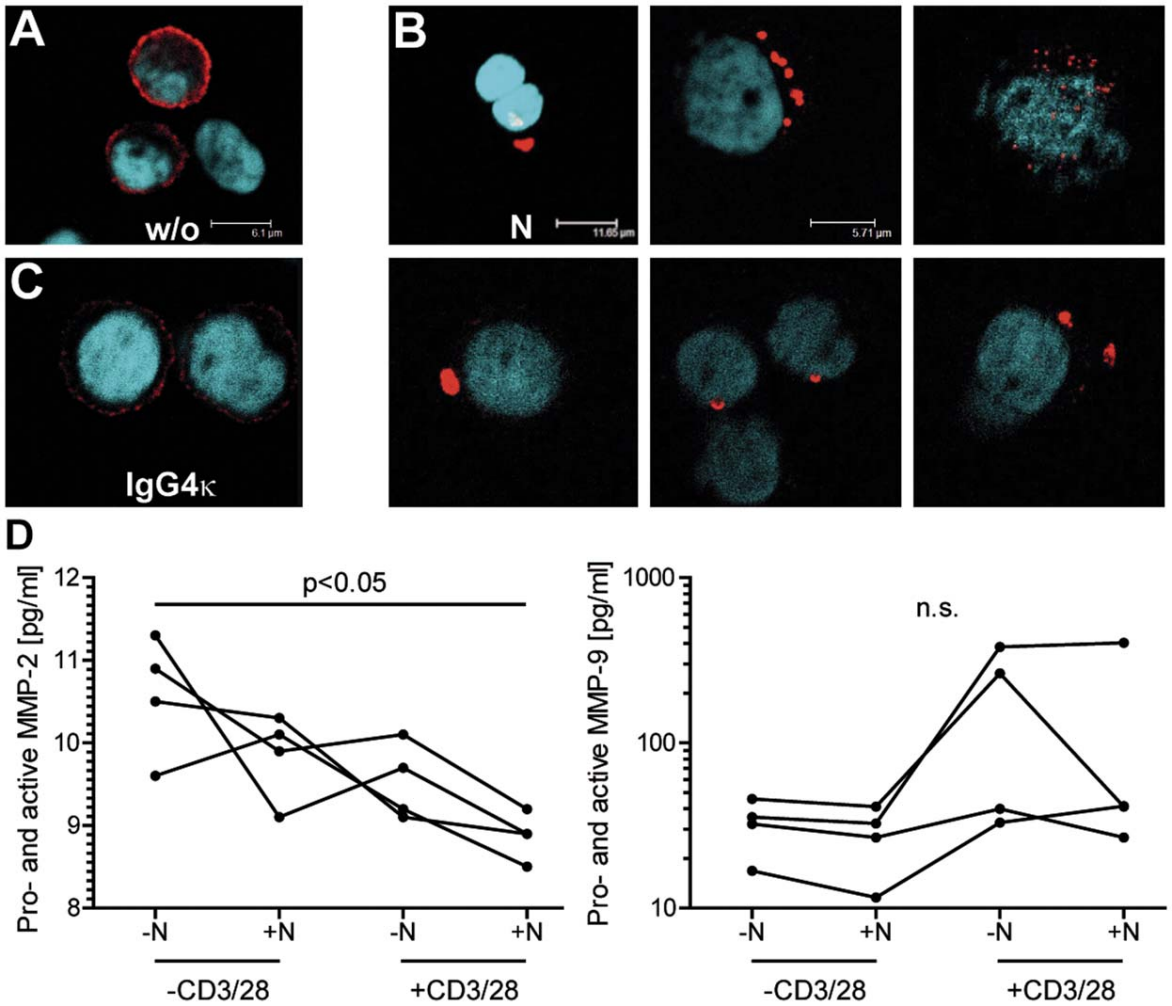
Integrin α-4 is subject to rapid internalization and degradation upon natalizumab binding. (A–C) Immunocytochemical staining of CD49d and counter-staining of nuclei in PBMC that were either left untreated (A), stimulated with 30 µg/ml natalizumab for 24 h (B) or stimulated with 30 µg/ml IgG4κ isotype control (C). Cells were fixed with 3.7% PFA and permeabilized with 0.1% Triton X-100 before staining. Representative of samples from 4 different donors. (D) PBMC were stimulated with anti-CD3/28 and/or natalizumab as indicated for 24 h. Subsequently, total protein levels of MMP-2 and MMP-9 were determined in cell culture supernatants by commercial ELISA kits. Friedman tests, followed by Dunn’s post tests were performed for statistical analysis.

We next sought to further investigate the fate of integrin α-4 after natalizumab binding. Immunocytochemical staining of CD49d in unstimulated PBMC allowed distinction of different lymphocyte subpopulations according to differential integrin α-4 expression levels, as observed by flow cytometry. CD49d mostly showed a fine granular pattern of membrane staining, with only minor intracellular staining observed under a confocal microscope (Figure 6A). Within 24 h, natalizumab induced a marked reduction of CD49d surface expression. Here, single cells showed CD49d-positive, speckled intracellular inclusions, while overall CD49d surface staining was clearly reduced. Examples of intracellular inclusions are shown in Figure 6B. In contrast, isotype control stimulations with IgG4κ did not show an effect on overall CD49d expression levels or subcelluar localization (Figure 6 C).

As only single cells displayed intracellular inclusions after only 24 h of natalizumab stimulation, while the vast majority of cells showed very low CD49d expression levels, we next evaluated potential CD49d secretion into cell culture supernatants. Of note, some members of the integrin family were described to be subject to shedding from the cell surface by metalloproteinases [34], and VLA-4 was recently discovered to be a docking site for matrixmetalloproteinase-9 (MMP-9) in leukemic albeit not in normal B cells [35]. Employing filter centrifugation for protein enrichment of PBMC culture supernatants and two different antibodies against the extracellular domain of CD49d, we were not able to detect CD49d or its potential fragments by Western blotting. For this, samples from 4 different donors had been tested in all combinations of CD3/28 and natalizumab stimulation for 24 h (data not shown). To corroborate this negative finding, we determined protein levels of MMP-2 and MMP-9 in PBMC culture supernatants. Surprisingly, natalizumab induced a modest reduction of MMP-2 secretion, as did CD3/28 costimulation in a synergistic manner (p<0.05), and it showed a trend towards a reduction of MMP-9 protein secretion, which in contrast to MMP-2 was strongly induced by CD3/28. Regarding the fate of CD49d after natalizumab binding, this set of experiments indicated rapid intracellular degradation upon internalization.

### Natalizumab Treatment Gives Rise to Immediate Upregulation of IL-2, IFN-γ and IL-17 in Some MS Patients

Although primary CD4^+^ T cells had been analyzed during the course of the experiments, it was not clear if autoimmune-active cells would react the same way. Therefore, PBMC were isolated from 9 RRMS patients immediately before and 24 h after the very first receipt of natalizumab. To avoid any perturbing influence from separation procedures, no CD4^+^ T cell isolation was performed. PBMC from both blood withdrawals were immediately analyzed or gently stimulated by plate-bound anti-CD3/28 for 24 h without any further addition of natalizumab. Therefore, any difference observed can be attributed to the infused drug.

**Figure 7:**
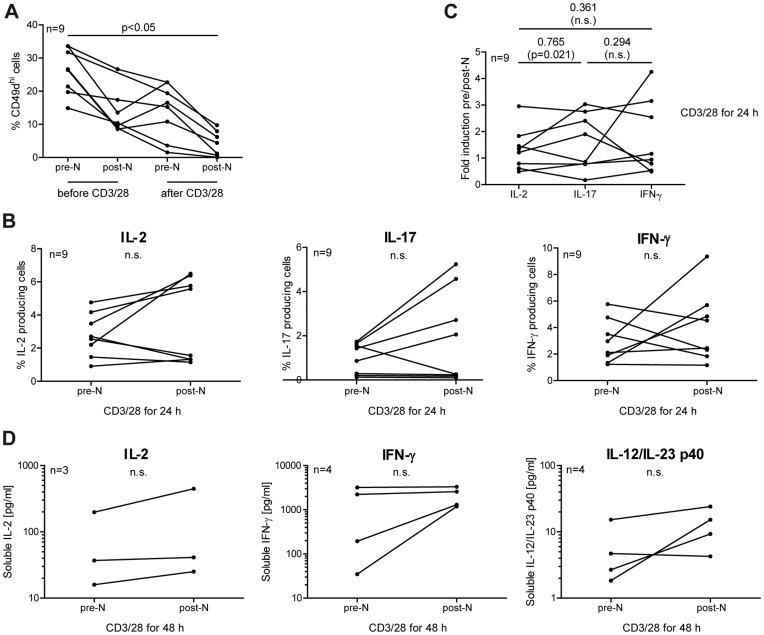
Natalizumab influences CD4^+^ T cells from MS patients immediately after the very first infusion. Pre- and post-natalizumab blood samples were taken from 9 patients with RRMS. (A-C) PBMC were cultivated with plastic-coated anti-CD3/28 for 24 h. (A) Cells were stained for surface expression of CD4 and CD49d before and after *in vitro* restimulation. (B, C) Cells were stained for intracellular IL-2, IL-17 and IFN-γ concomitantly with staining for surface CD4, and analyzed by FACS. (D) as in A-C, but *in vitro* restimulation for 48 h. Secretion of interleukins was evaluated by cytometric bead arrays. A two-tailed Kruskal-Wallis test followed by Dunn’s posttest was used in (A) for statistical analysis (no matched pairs test possible due to one missing value). Two-tailed Wilcoxon matched-pairs signed rank tests were performed for statistical analysis in (B) and (D). Spearman correlation coefficients were calculated in (C).

In accordance with the findings in CD4^+^ T cells from healthy donors, which were loaded with natalizumab *in vitro*, PBMC from MS patients partially lost CD49d/VLA-4 integrin from their surface within 24 h after infusion. Consistent with the *in vitro* experiments using Jurkat and CD4^+^ T cells from healthy donors, (re-) stimulation engaging the TCR and costimuli led to a marked reduction of surface CD49d, which was even more pronounced after the patients had received natalizumab (Figure 7A). Furthermore, intracellular staining revealed an increase of IL-2, IL-17 and IFN-γ in some of the patients within this short period of treatment with natalizumab. Due to inter-individual variability, however, it did not reach statistical significance in the cohort as a whole when each of the individual cytokines was evaluated. Of interest, IL-2 and IL-17 positive cell counts rose in those patients who had higher positive cell numbers at baseline, possibly corresponding to stronger Th17 activation before starting natalizumab therapy. The opposite was observed for IFN-γ/Th1: low pre-treatment cell numbers were increased, while higher numbers were decreased by natalizumab (Figure 7B). Spearman analysis demonstrated a strong intra-individual correlation between IL-2 and IL-17 induction (R = 0.765) after natalizumab infusion, while no significant correlation between these two cytokines and IFN-γ was observed (Figure 7C). This argued for a defined direct natalizumab effect on cytokine secretion in a subset of patients. Cytometric bead arrays were performed with some of the samples. Results demonstrated enhanced secretion of IL-2 (2-fold) and IFN-γ (3-fold) after 48 h of *in vitro* restimulation with anti-CD3/28 in the majority of tested patients. IL-17 and IL-21 concentrations were too low to detect, as IL-4 and IL-10 levels were unchanged. Interestingly, however, IL-12/IL-23 p40 clearly exhibited fortified secretion when blood was drawn from natalizumab-infused patients, suggesting an additional, but indirect effect of natalizumab on CD4^+^ T cells to express IFN-γ and IL-17 (Figure 7D). In conclusion, CD4^+^ T cells from a subset of MS patients newly starting natalizumab treatment appeared to exhibit a drug-induced pro-inflammatory phenotype within the first 24 h after treatment initiation.

## Discussion

Natalizumab blocks the firm arrest of leukocytes to the endothelium of the BBB, preventing their extravasation into the CNS with high efficacy. Here we demonstrate that this mAb – named Tysabri® as a drug – additionally has a direct costimulatory effect on T cells, mildly supporting a pro-inflammatory phenotype. This was not only shown *in vitro* with CD4^+^ T cells from healthy donors, but also evident in the peripheral blood of MS patients within 24 h after the very first infusion of natalizumab.

Natalizumab exerts its leukocyte adhesion blocking effect by direct molecular interference with the binding of VLA-4 on leukocytes to VCAM-1 on CNS microvascular endothelium. In addition, integrin α-4 was found to be partially but persistently downregulated on PBMC of MS patients receiving the drug, potentially representing an additional mechanism of adhesion blockade [36], [37]. Our data now prove that the downregulation of CD49d surface expression on T lymphocytes is a direct and rapid consequence of natalizumab binding. Consistent with another blocking mAb against VLA-4, which mediated the internalization of integrin α-4 on rat neutrophils [38], we observed natalizumab to cause intracellular aggregates of CD49d, while no CD49d was detected in cell culture supernatants. In agreement, we observed MMP-2 and MMP-9 being downregulated rather than increased by natalizumab, excluding shedding of VLA-4 as found for integrin β2 from macrophages [34]. Therefore, natalizumab probably causes internalization of VLA-4.

However, extravasation is only one possible process that is mediated by CD49d and its integrin binding partners β-1 and β-7. Several cellular events are affected in addition to adhesion and migration, including cell differentiation, polarization, activation and survival [8]. Therefore, we explored possible immediate signaling capabilities of natalizumab, addressing the status of activation and differentiation of CD4^+^ T cells in the peripheral blood, which are excluded from the CNS during natalizumab treatment. And indeed, we observed natalizumab-mediated signaling, which mildly raised the levels of pro-inflammatory lymphokines. Here our finding of CD49d reduction depending on the degree of T cell activation provides one possible explanation why natalizumab exerted a stronger relative effect on cytokine secretion in partially vs. fully activated T cells.

Evidence that integrin α-4/β-1 is involved in priming of naive T cells has been provided: upon binding of α-4 activating antibodies, VLA-4 colocalizes with the signaling molecules of the TCR complex and T cells are shifted towards a Th1-type immune response [39]. Signaling was suggested, as α-4/β-1 localizes within the peripheral supramolecular activation cluster (pSMAC) of synapses between T cells and dendritic cells/B cells [39]. Because integrins link the cytoskeleton with the extracellular environment, engagement of VLA-4 sustains signaling by altering the dynamics of actin filaments and signaling molecules at the immunological synapse [40]. However, so far it had not been demonstrated that also the adhesion-blocking antibody natalizumab acts as a costimulus for T cells, very much resembling α-4/β-1 activating signals. Integrin-mediated cellular activities involve the activation of intracellular signaling pathways associated with focal adhesion kinase (FAK). FAK directly binds to the cytoplasmic domain of integrin β-1, but also interacts with CD4 [41]. FAK-mediated pathways include the activation of ERK, providing survival signals [42], and while α-4/β-1 costimulation of human T cells was recently found to enhance ERK phosphorylation as well as IL-2 and IFN-γ production [43], we now provide evidence that natalizumab signals via the same route. Therefore, when antigen cross-links the TCR this leads to NFAT as well as integrin (‘inside-out’ signal) activation; then natalizumab supports nuclear AP1 induction via MAPK-signaling. NFAT and AP1 form the crucial transcription factor complex for lymphokine expression [44]. Depending on the context of activation, i. e. classical costimuation via CD28 family members and present cytokines as well as possible former encounters of the T cell, integrin signaling might be enough to support a pro-inflammatory differentiation of the T cell. The observed consistent increase in FOXP3 expression probably reflects activation of human effector T cells, where neither proliferation nor cytokine production are repressed by FOXP3 [33]. In case FOXP3 regulatory T cells would be induced or propagated by more IL-2 present [45], they may not be able to repress rebound disease activity [46].

Prolonged therapeutic CD49d blockade by natalizumab certainly results in unexpected changes of the immune system. Enrichment with leukocytes of different origin in the periphery and mobilization of hematopoietic stem/progenitor cells [47], [48], [49], enlargement of circulating TNF-α^+^, IFN-γ^+^, and IL-17^+^ cell numbers [28], [29], [30], [50], [51], and altered gene expression profiles [52] were reported. So far this was interpreted as defective stem cell homing into the bone marrow and as sequestration of activated cells in the peripheral circulation. Based on our data it can be concluded, though, that in addition costimulatory features mediated by natalizumab support a pro-inflammatory phenotype in the periphery. These primary signaling events via VLA-4 should funnel into positive feed-back loops with cross-talks to other cell types, explaining the robust long-term pro-inflammatory bias observed [28], [29], [30]. That Th1 differentiation is a specific feature of natalizumab was demonstrated by a comparative study with glatiramer acetate just recently [53].

Overall, this helps to explain a rebound of disease activity observed in some patients after natalizumab cessation [24], [25], [26]. One current case report not only clearly indicates that such a relapse after natalizumab withdrawal can be mortal but additionally that it is primarily mediated by CD4^+^ T cells [27]. Therefore, the inter-individually different and less dramatic primary events detected in this study may initiate a fulminant phenotype laying ground for severe rebound disease in rare cases.

Furthermore, PML-IRIS in natalizumab-treated patients [19], [20], [21], [22] might be partially attributable to pro-inflammatory effects of natalizumab outside the CNS: JC virus-specific T cells are pre-activated, enlarged in number, and when entering the CNS not efficiently controllable by regulatory T cells or other mechanisms. PML-IRIS also occurs in other situations of immune reconstitution, such as in HIV patients starting highly active anti-retroviral therapy (HAART). However, while the overall clinical outcome of HAART-associated IRIS was found to be favourable in the majority of HIV patients with PML [54], natalizumab-associated IRIS in MS patients with PML often has a poor outcome [19], [21]. This difference might at least partially be due to the presence of dysregulated CNS-autoreactive T cells in natalizumab-treated MS, but not in HIV patients.

Taken together, natalizumab efficiently excludes activated lymphocytes from the CNS by at least two mechanisms, but the activated pro-inflammatory situation in the periphery caused by direct signaling capacities of natalizumab might help to explain some adverse effects of this treatment. Novel integrin α-4 blocking pharmacological agents, which are currently in clinical development, should be carefully evaluated for their signaling potential.
